# Validation of the clinical applicability of knowledge‐based planning models in single‐isocenter volumetric‐modulated arc therapy for multiple brain metastases

**DOI:** 10.1002/acm2.13022

**Published:** 2020-09-19

**Authors:** Noriko Kishi, Mitsuhiro Nakamura, Hideaki Hirashima, Nobutaka Mukumoto, Keiichi Takehana, Megumi Uto, Yukinori Matsuo, Takashi Mizowaki

**Affiliations:** ^1^ Department of Radiation Oncology and Image‐Applied Therapy Graduate School of Medicine Kyoto University Kyoto Japan; ^2^ Department of Information Technology and Medical Engineering Division of Medical Physics Graduate School of Medicine Human Health Sciences Kyoto University Kyoto Japan

**Keywords:** k‐fold cross‐validation, knowledge‐based planning, multiple brain metastases, single‐isocenter VMAT

## Abstract

**Purpose:**

To validate the clinical applicability of knowledge‐based (KB) planning in single‐isocenter volumetric‐modulated arc therapy (VMAT) for multiple brain metastases using the *k*‐fold cross‐validation (CV) method.

**Methods:**

This study comprised 60 consecutive patients with multiple brain metastases treated with single‐isocenter VMAT (28 Gy in five fractions). The patients were divided randomly into five groups (Groups 1–5). The data of Groups 1–4 were used as the training and validation dataset and those of Group 5 were used as the testing dataset. Four KB models were created from three of the training and validation datasets and then applied to the remaining Groups as the fourfold CV phase. As the testing phase, the final KB model was applied to Group 5 and the dose distributions were calculated with a single optimization process. The dose‐volume indices (DVIs), modified Ian Paddick Conformity Index (mIPCI), modulation complexity scores for VMAT plans (MCSv), and the total number of monitor units (MUs) of the final KB plan were compared to those of the clinical plan (CL) using a paired Wilcoxon signed‐rank test.

**Results:**

In the fourfold CV phase, no significant differences were observed in the DVIs among the four KB plans (KBPs). In the testing phase, the final KB plan was statistically equivalent to the CL, except for planning target volumes (PTVs) D_2%_ and D_50%_. The differences between the CL and KBP in terms of the PTV D_99.5%_, normal brain, and D_max_ to all organs at risk (OARs) were not significant. The KBP achieved a lower total number of MUs and higher MCSv than the CL with no significant difference.

**Conclusions:**

We demonstrated that a KB model in a single‐isocenter VMAT for multiple brain metastases was equivalent in dose distribution, MCSv, and total number of MUs to a CL with a single optimization.

## 1 Introduction

Brain metastases are the most common intracranial tumors, which are present in approximately 2% of cancer patients at the time of the primary diagnosis, with a prevalence ranging from 15% (small cell lung cancer) to < 0.1% (prostate cancer) and a median overall survival of less than 1 yr.^1^ The incidence of brain metastases is observed to increase because of the prevalence of magnetic resonance imaging (MRI), which has improved the rates of detection. Moreover, the development of systemic therapy has improved the survival after the primary diagnosis of brain metastases. The standard treatment strategy for multiple brain metastases is whole‐brain radiotherapy (WBRT); however, WBRT leads to the deterioration of neurocognitive function and patient quality of life.^2^

Stereotactic radiosurgery (SRS) is an irradiation technique that requires the precise fixation of the patient, localization of the target, and the application of highly biologically effective radiation doses.^3^ Using SRS, the irradiated dose to the normal brain can be reduced, and re‐irradiation can be considered even in post‐SRS patients who experience intracranial recurrence. In patients with 1–4 brain metastases, SRS causes lesser neurocognitive deterioration compared to SRS plus WBRT; additionally, there is no difference in the overall survival between the SRS and SRS plus WBRT.^4^, ^5^, ^6^ Although the clinical advantage of SRS over WBRT is controversial in patients with more than four brain metastases, SRS is considered an effective and safe treatment option, especially in patients with a favorable prognosis.^7^

Linac‐based single‐isocenter volumetric‐modulated arc therapy (VMAT) can accomplish clinically equivalent dose distributions to gamma knife radiosurgery, but with a reduced delivery time.^8^ However, the treatment planning of single‐isocenter VMAT for multiple brain metastases is generally time‐ and resource‐intensive. When performing the optimization, planners manually determine the optimization parameters; nevertheless, it is cumbersome to identify the optimization parameters due to the presence of multiple target volumes with different sizes and characteristics. In addition, for one target located adjacent to another in the same plane, several optimizations are required to minimize the dose spillage between the target volumes; as there is no clearly defined goal, the process can seem endless.

Recently, knowledge‐based (KB) planning has become available for clinical use as a tool assisting inverse planning.^9^ KB planning uses databases constructed according to the anatomical positions and doses of previously treated patients and establishes a machine‐learned model for dose‐volume histogram (DVH) estimation. The model estimates DVHs to create treatment plans for new patients. A recently published review paper showed that, generally, KB models produce plans of comparable quality to those of expert planners, while also reducing the time and effort required to generate plans for various disease sites.^9^

When verifying machine‐learned models, the predictive performance of the models should be evaluated on unseen data.^10^ The hold‐out validation method is a simple approach for model evaluation where the data are categorized into two subsamples: training and testing. However, this method is prone to subsample bias and is inadequate for small sample sizes. The *k*‐fold cross‐validation (CV) method is another technique for the evaluation and comparison of machine‐learned models. The *k*‐fold CV method splits the data into *k* equal sized subgroups; one subgroup is used as a validation group and the remaining subgroups are used as a training dataset. With *k*‐fold CV, the whole dataset can be used for both training and validation, and this method is affected by pessimistic bias in a lesser manner, compared to the hold‐out method. In general, *k*‐fold CV is usually considered as the preferred method because it allows the model to train via multiple train‐test splits providing a better indication of how well the model will perform on unseen data. Most studies on KB planning employed the hold‐out method for prostate/uterine cancer, head and neck cancer, and lung, liver, and primary brain tumors.^11^, ^12^, ^13^, ^14^, ^15^ To date, however, there has been insufficient literature comparing the performance of KB models through *k*‐fold CV, or on the application of KB planning to multiple brain metastases.

The purpose of this study was to validate the clinical applicability of KB planning in single‐isocenter VMAT for multiple brain metastases using the *k*‐fold CV method.

## 2 Methods

### 2.1 Patients

This study included 60 consecutive patients with multiple brain metastases treated using single‐isocenter VMAT (28 Gy in five fractions) in our institute from October 2015 to December 2018. The study participants included 29 men and 31 women, with a median age of 64 yr (range: 26–88 yr). In total, there were 317 multiple brain metastases. The patients were divided equally and randomly into five groups (Groups 1–5) using Python scripts (Python version 3.7).

This study was performed in accordance with the Declaration of Helsinki (1975, as revised in 2013). Written informed consent was obtained from all patients before initiating radiotherapy and the Institutional Review Board of the Kyoto University Hospital approved this study (R1446).

### 2.2 Treatment planning

On the day of CT, the patient’s head was immobilized with a thermoplastic mask in the supine position. Contrast‐enhanced computed tomography (CECT) scans were acquired with the following imaging parameters: a slice thickness of 1 or 1.25 mm (depending on the CT scanner; SOMATOM Definition AS; Siemens Medical Systems, Erlangen, Germany, and LightSpeed RT16; General Electric Medical Systems, Waukesha, WI, USA, respectively), a pixel matrix of 512 × 512 pixels, and a field of view of 400 mm. CECT was combined with gadolinium‐enhanced T1‐weighted MRI with the images obtained within 1 month prior to the CT simulation. The gross tumor volume (GTV) was defined as the visible lesion on both the CT and MRI; no clinical target volume was defined. Planning target volumes (PTVs) were defined by adding a margin of 1–2 mm to the GTV. The median number of PTVs present in any plan was 4 (range: 2–18). The organs at risk (OARs) included the normal brain (whole brain minus the PTVs), brainstem, eyes, lens, optic nerves, chiasm, and skin (defined as a structure cropped 5 mm from the body).

The isocenter was located at the center of all the PTVs. The VMAT plans were created using 3–5 arcs, including 1 full coplanar arc and 2–4 non‐coplanar partial arcs with a couch angle of ± 60°. The collimator angle was manually selected depending on the size and location of the target. Photon beam energies of six flattening filter‐free (FFF) and 10 FFF MV photon beams were used. All the treatment plans were generated the Eclipse planning system (version 13; Varian Medical Systems, Palo Alto, CA, USA), delivered from a TrueBeam STx instrument with a high‐definition 120‐leaf multileaf collimator (MLC) (Varian Medical Systems). Dose calculations were performed using Acuros XB (version 13.7; Varian Medical Systems) with heterogeneity correction and a 1‐mm grid resolution.

Plan normalization was performed in a manner such that at least 99.5% of the prescribed dose (D_99.5%_) of 28 Gy (five fractions) was generally delivered to each PTV. Plan optimization was performed so that the near‐maximum dose (D_2%_) to all PTVs was around 40 Gy (Per protocol, 135–150%) and the irradiated dose to the OARs was as low as possible.

### 2.3 Model creation, evaluation, and selection

**Fig. 1:**
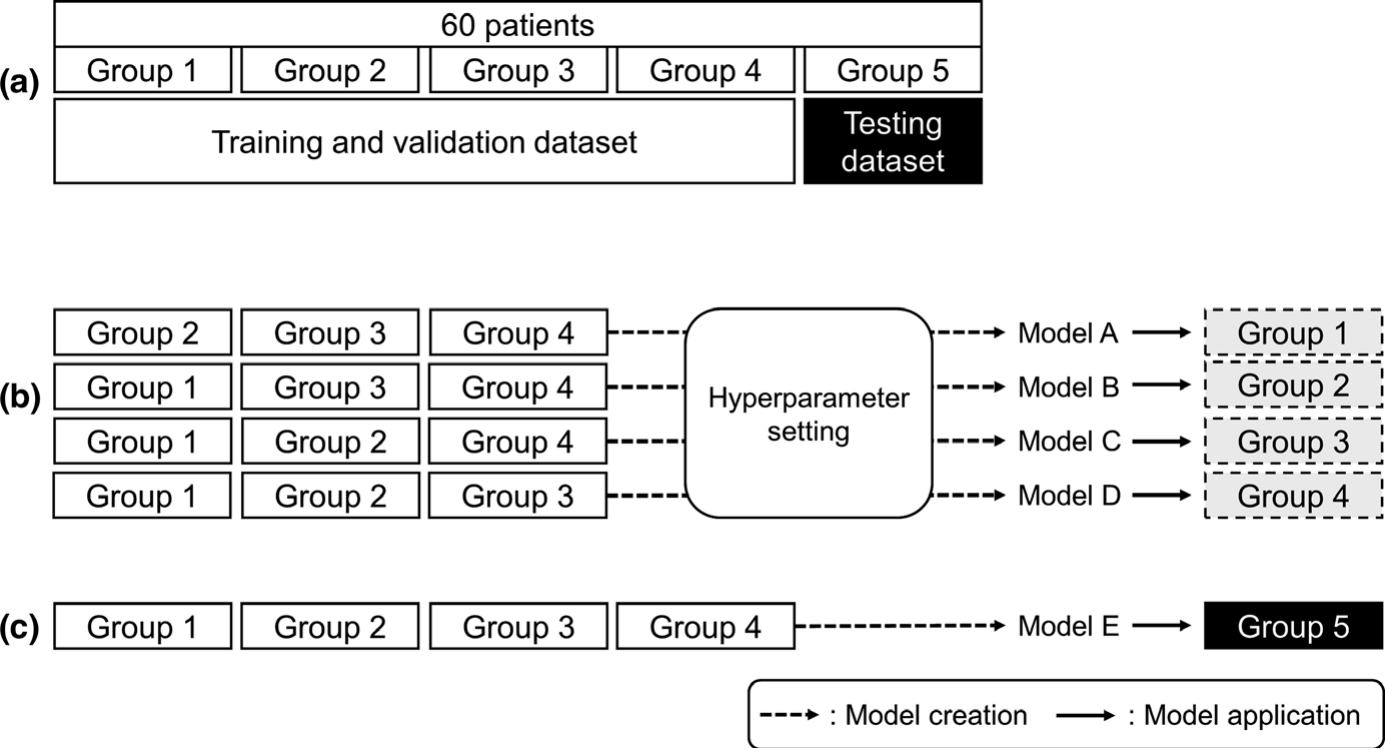
Schematic of the fourfold cross‐validation (CV) method; (a) dataset splitting, (b) hyperparameter setting of dose‐volume histogram (DVH) estimation models created from three subgroups (Models A ~ D) and model application to the remaining subgroup, and (c) model created based on all training and validation datasets (Model E), and application thereof to the testing dataset. Broken arrows denote model creation and solid arrows denote model application in (b) and (c).

The fourfold CV method was used for model creation, evaluation, and selection (Fig. 1). First, the dataset was split into two; Groups 1–4 were used as the training and validation dataset and Group 5 as the testing dataset (Table 1). Next, the model configuration and hyperparameter setting were conducted using RapidPlan^TM^ (version 13.7; Varian Medical Systems).^16^ RapidPlan^TM^ is a machine learning system based on the geometric relation of the structures and DVH. As part of the model‐creating process, the dosimetric and geometric data were extracted from the database to establish DVH estimation models using regression techniques. In RapidPlan^TM^, there are mainly two types of optimization objectives; a fixed objective and a line objective. The former manually adds a fixed upper or lower dose or volume, with a fixed priority or with a generated priority created by the DVH estimation algorithm. Fixed objectives can be used for both OARs and target structures. On the other hand, the latter generates an estimated DVH range only for the OARs, which is also created by the DVH estimation algorithm automatically. Using a line objective, the optimization is performed so that the OAR will receive a dose as low as the estimated DVH range. Therefore, the volumes, doses, and priorities are automatically generated in a line objective.

**Table 1:** Summary of each Group. The patients were divided randomly into five groups (Groups 1–5). The data of Groups 1–4 were used as the training and validation dataset and those of Group 5 were used as the testing dataset.

Group	Role	Total number of PTVs	Number of PTVs per patient [Median (min–max)]	Size (cm^3^) [Median (min–max)]
1	Training/Validation	70	6 (2–12)	0.6 (0.1–12.3)
2	Training/Validation	57	3 (2–13)	0.6 (0.1–16.9)
3	Training/Validation	60	5 (2–14)	0.4 (0.1–12.9)
4	Training/Validation	62	4 (2–12)	0.5 (0.1–10.2)
5	Testing	68	4 (2–18)	0.5 (0.2–13.5)

*Abbreviation*: PTV, planning target volume.

Subsequently, four DVH estimation models were created from the training and validation dataset with the determined hyperparameter setting shown in Table 2: Model A (MA; Groups 2–4), Model B (MB; Groups 1, 3, and 4), Model C (MC; Groups 1, 2, and 4), and Model D (MD; Groups 1–3). MA, MB, MC, and MD were applied to the remaining of the training and validation dataset. In the validation phase, the generalization ability was assessed, which means that the model from training data can reproduce an acceptable outcome when applied to unseen data. The dose‐volume indices (DVIs) were compared among four KB‐generated plans (KBP‐A1: MA applied to Group 1; KBP‐B2: MB applied to Group 2; KBP‐C3: MC applied to Group 3; and KBP‐D4: MD applied to Group 4). A lack of any statistical difference among the DVIs of the four KB plans (KBPs) and a D_2%_ for all PTVs ranging from 130% to 155% of the prescribed dose (clinically acceptable variation), was taken to indicate that the models were not overfitted and had a good generalization performance.

**Table 2:** Optimization objectives used in the model.

Structure	Type	Volume (%)	Dose (cGy)	Priority
Brain‐PTVs	Line	Generated	Generated	Generated
Brainstem	Upper	Generated	1000	Generated
	Line	Generated	Generated	Generated
Eyes	Upper	Generated	150	Generated
	Line	Generated	Generated	Generated
Lens	Upper	Generated	100	Generated
Optic nerves	Upper	Generated	200	Generated
	Line	Generated	Generated	Generated
Skin	Line	Generated	Generated	Generated
PTV	Upper	0	3950	300
	Upper	10	3850	Generated
	Upper	30	3650	Generated
	Upper	50	3450	Generated
	Upper	70	3300	Generated
	Upper	90	3050	200
	Lower	0.5	3900	200
	Lower	10	3800	Generated
	Lower	30	3600	Generated
	Lower	50	3400	Generated
	Lower	70	3250	Generated
	Lower	90	3000	Generated
	Lower	99.5	2900	Generated
	Lower	100	2850	Generated

Note

The line objective is a type of optimization objective and the values of volume, dose, and priority are automatically generated by RapidPlan^TM^. Priorities are also generated in this study with respect to some upper or lower objectives.

Abbreviations: Line, line objective; Lower, lower objective; PTV, planning target volume; Upper, upper objective.

Finally, in the testing phase, the accuracy of the created models was focused upon. Model E (ME; Groups 1–4) was constructed using all the training and validation datasets and the same hyperparameter settings as shown in Table 2. ME was applied to Group 5 (independent testing dataset), and a KBP (KBP‐E5) was then generated. A lack of statistical difference between the DVIs extracted from the KBP‐E5 and the CL of each corresponding PTV and each OAR in the same patient was regarded to indicate that the KB models yielded clinically acceptable plans.

### 2.4 KB‐generated plan optimization and dose calculation

One optimization cycle was performed for each model without the modification of the KB‐generated optimization objectives. Thereafter, the dose distribution was calculated with the same beam arrangement used in the CL and plan normalization was performed.

### 2.5 Evaluation indices

In the validation phase, the DVIs for the PTV (D_2%_, D_50%_, and D_99.5%_), normal brain (V_5 Gy_, V_10 Gy_, V_14 Gy,_ V_20 Gy_, and V_28 Gy_), and the maximum dose (D_max_) to all OARs were compared among plans.

In the testing phase, in addition to the DVIs above, the Ian Paddick Conformity Index (IPCI) was also employed to evaluate the dose distributions.^17^ The definition of the IPCI is [TV_PIV_]^2^/ [TV × PIV], where the TV_PIV_ is the target volume covered by the prescription dose, TV is the target volume, and PIV is the prescription isodose volume. For multiple targets, the modified IPCI (mIPCI) was defined as ([TV_PIV_sum_]^2^/ [TV_sum_ × PIV_sum_]), where TV_PIV___sum_ is the summed target volumes enclosed by an isodose line of the prescription dose, TV_sum_ is the summed volume of all PTVs, and PIV_sum_ is the summed prescription isodose volume. The mIPCI had values in the range 0–1, with the scores approaching 1 when PTVs were conformally covered with isodoses of the prescription dose. The modulation complexity scores for the VMAT plans (MCSv) and the total number of monitor units (MUs) were used as additional metrics. The MCSv was calculated based on the leaf sequence variability (LSV) parameter and aperture area variability.^18^ The LSV was defined for each control point considering in each bank the differences in position between adjacent MLC leaves. The MCSv demonstrated values in the range 0–1; additionally, the scores decreased when the modulation increased.

### 2.6 Statistical analysis

In the validation phase, the Friedman test was performed on the DVIs of all the PTVs and OARs to determine if there is any difference between the KBP‐A1, B2, C3, and D4, followed by the unpaired Wilcoxon signed‐rank test with Bonferroni correction for multiple comparisons as a post hoc test. Meanwhile, in the testing phase, a paired Wilcoxon signed‐rank test was used to evaluate the significant differences between the CL and KBP‐E5 for each corresponding PTVs and OAR in the same patient. The statistical significance was set at *P* < 0.05. All statistical analyses were performed using R software (version 3.5.1; R Development Core Team, Vienna, Austria).

## 3 Results

### 3.1 Validation phase

**Fig. 2:**
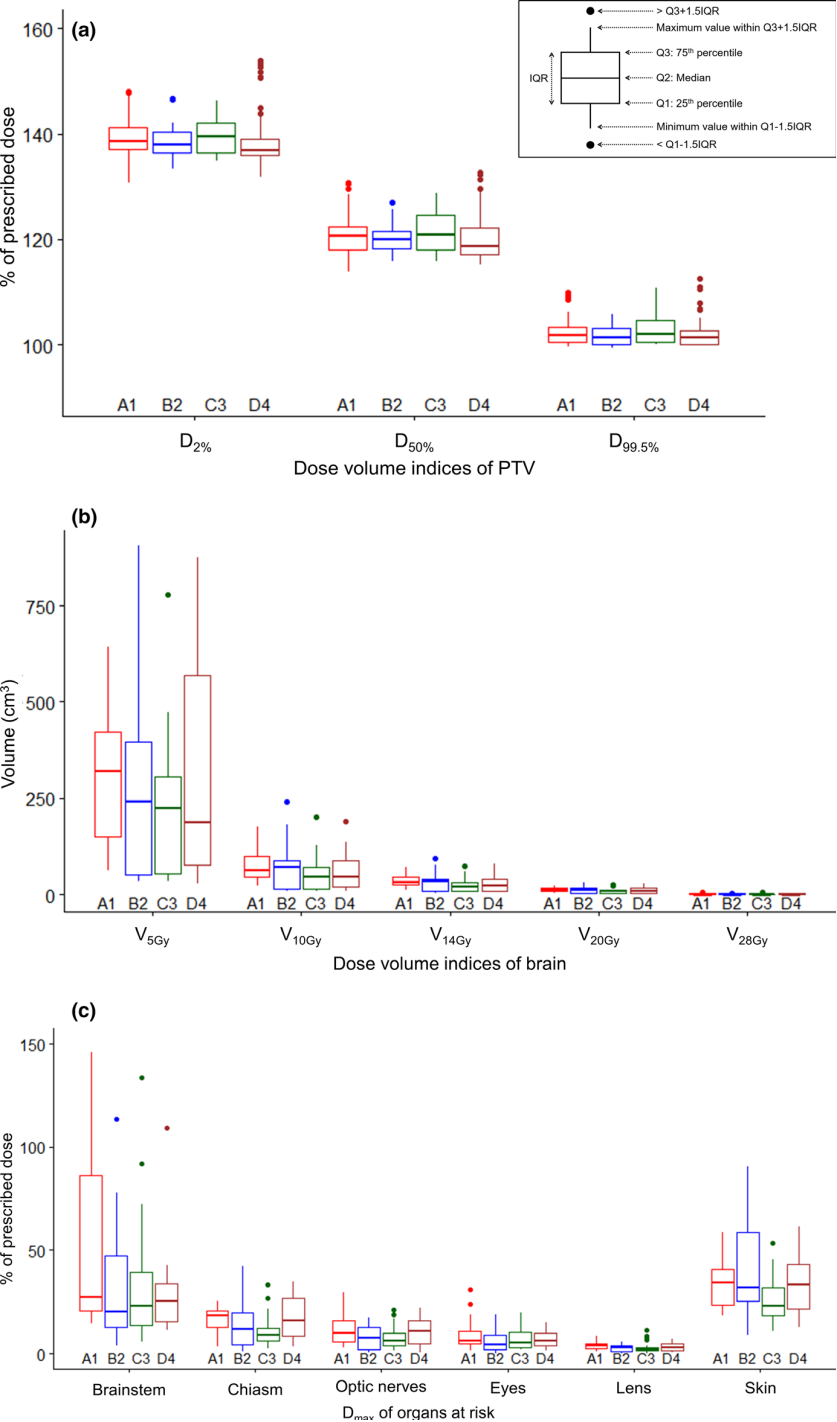
The box‐and‐whisker plots of the dose‐volume indices (DVIs) of (a) the planning target volume (PTV), (b) normal brain, and (c) other organs at risk (OARs): comparison of KBP‐A1, B2, C3, and D4 in the fourfold CV phase. The boxes represent the interquartile range (IQR). Low outliers are below the first quartile minus 1.5 × IQR and high outliers are above the third quartile plus 1.5 × IQR. D_XX%_ = the dose to XX% of the target volume, V_XXGy_ = the volume receiving XX Gy, D_max_ = the maximum dose to the volume.

In the fourfold CV phase, no significant difference was observed in any DVI among the four KBPs. The details of the DVIs for all the PTVs, normal brain, and other OARs between the four KB models are shown in Fig. 2. The PTV D_2%_ values ranged from 130.8% to 153.9% of the prescribed dose, within clinically acceptable variations; thus, it was confirmed that the KB model was not overfitted and had a good generalization performance.

### 3.2 Testing phase

**Fig. 3:**
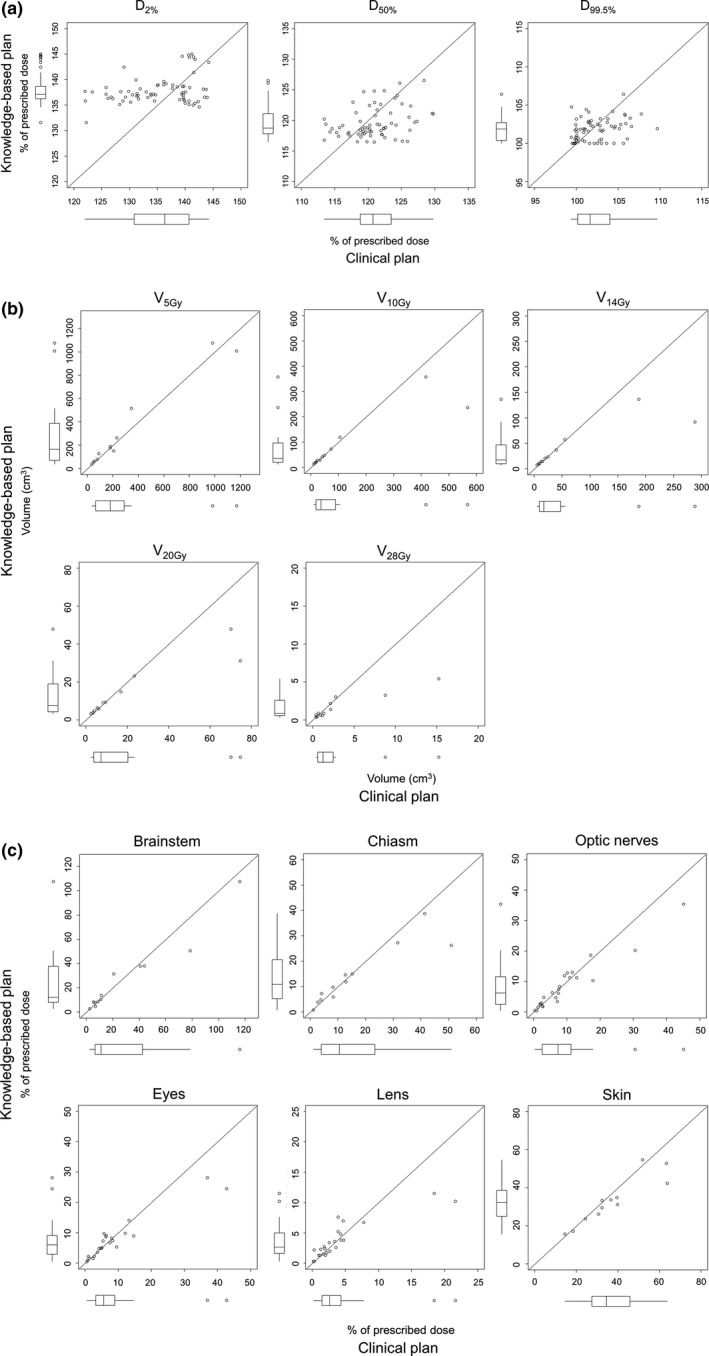
The scatter plots and box‐and‐whisker plots of the DVIs of (a) the PTV, (b) normal brain, and (c) other OARs for clinical plan (CL) and KBP‐E5 in the testing phase can be observed. The variation of the PTV in the KBP‐E5 was smaller than that of PTV in the CL. The plots of all the OARs were along or below the diagonal line (*y* = *x*). Abbreviations are the same as in Fig. 2.

In the testing phase, the D_2%_ of each PTV in KBP‐E5 (median, 137.2% [interquartile range (IQR), 2.4%]) was significantly higher than that of the corresponding PTVs in the CL (median, 136.3% [IQR, 9.6%]) (*P* = 0.005). The D_50%_ of each PTV in KBP‐E5 was significantly lower than that of the corresponding PTVs in the CL (median, 118.8% vs. 120.7% [IQR, 3.2% vs.4.6%]) (*P* = 0.02). The differences of each PTV D_99.5%_, normal brain (V_5 Gy_, V_10 Gy_, V_14 Gy,_ V_20 Gy_, and V_28 Gy_), and D_max_ to all OARs, were not significant in terms of the CL and KBP‐E5 (*P *> 0.05). Figure 3 shows the scatter plots of the DVIs of the PTV, normal brain, and other OARs for CL and KBP‐E5.

**Fig. 4:**
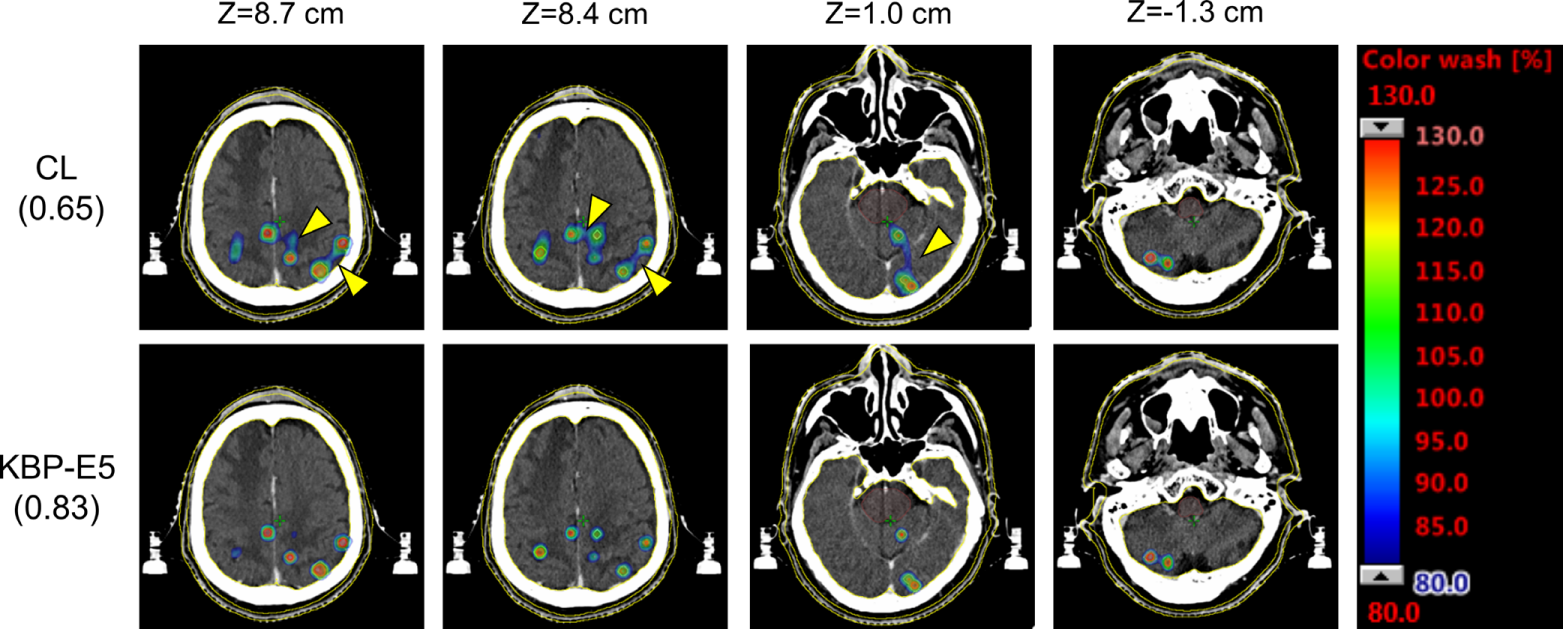
The representative dose distributions of the CL and the knowledge‐based plan (KBP) for a patient with 16 PTVs. The modified Ian Paddick Conformity Index (mIPCI) is shown in parentheses. The high‐dose spillage between the target volumes was minimized, as shown by yellow arrows.

**Fig. 5:**
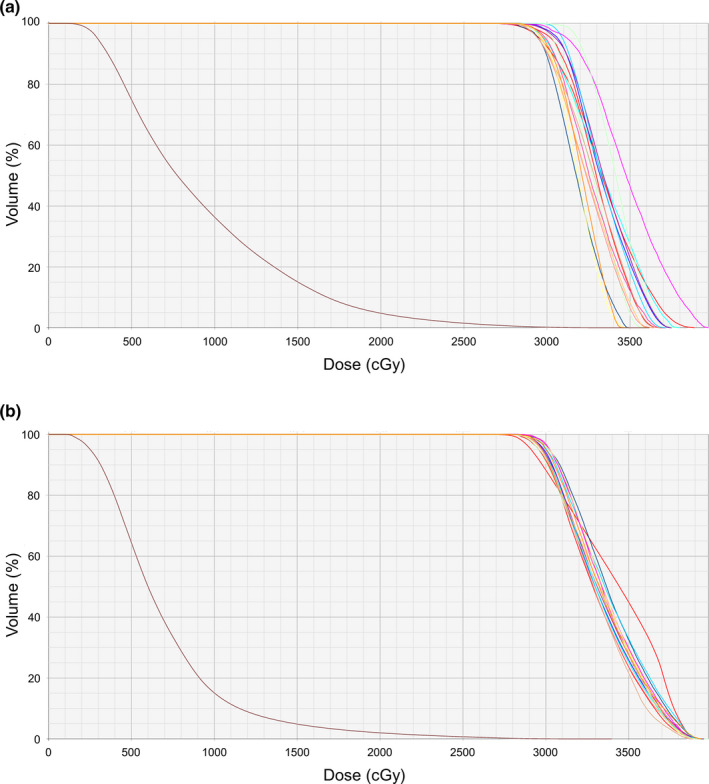
The representative DVHs of (a) the CL and (b) KBP‐E5 for a patient with 16 PTVs; the brain PTVs are shown in brown. The dose to the OARs was decreased and the DVHs for each PTV were more uniform for KBP‐E5 than for the CL.

The representative dose distributions of the CL and KBP‐E5 are shown in Fig. 4. KBP‐E5 minimized the dose spillage between the target volumes. The representative DVHs of the CL and KBP‐E5 are shown in Fig. 5; the irradiated dose to the OARs was decreased and the DVHs for each PTV was more uniform for KBP‐E5 than for the CL.

**Fig. 6:**
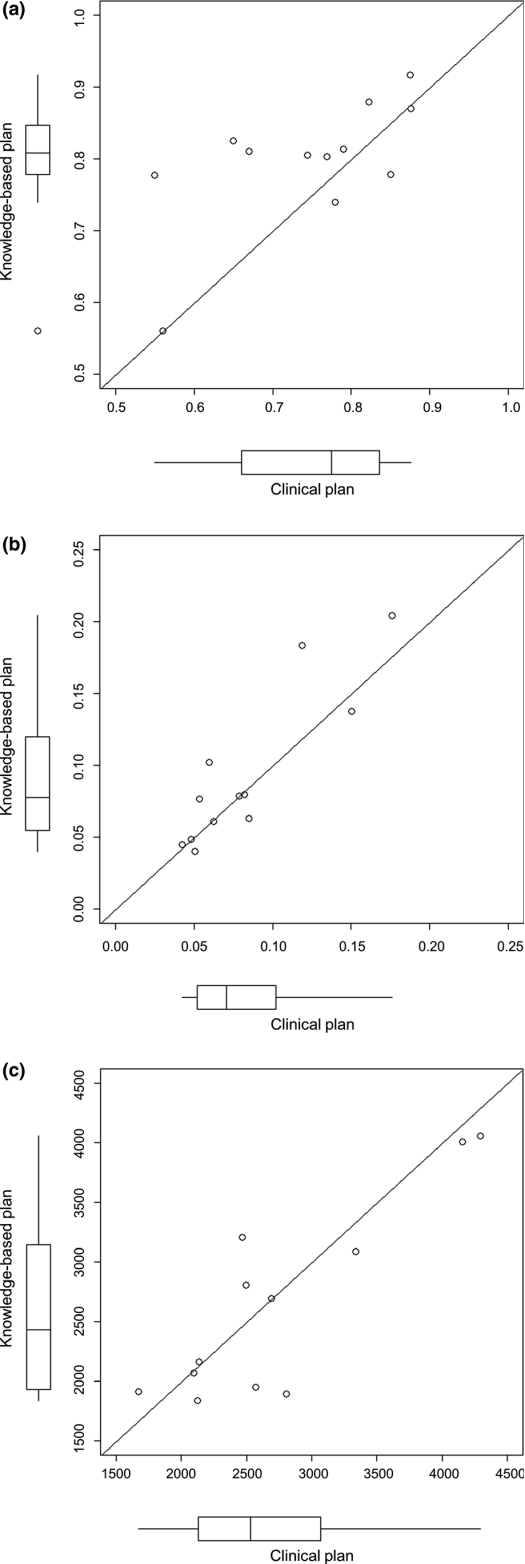
The scatter plots and box‐and‐whisker plots of (a) the mIPCI, (b) modulation complexity scores for VMAT plans (MCSv) and (c) monitor units (MUs) in the testing group. The scatter plots of mIPCI and MCSv were above the diagonal line (*y* = *x*). mIPCI = modified Ian Paddick Conformity Index. *Note:* The mIPCI was defined as ([TV_PIV_sum_]^2^/ [TV_sum_ × PIV_sum_]); TV_PIV_sum_ = the sum of target volumes enclosed by an isodose line of the prescription dose, TV_sum_ = the sum of volume of all PTVs, PIV_sum_ = the sum of prescription isodose volume. The mIPCI approaching 1 means that PTVs were conformally covered with the prescribed dose.

Figure 6 shows the scatter plots of the mIPCI, MCSv, and the total number of MUs for the CL and KBP‐E5. The scatter plots of mIPCI and MCSv were above the diagonal line (*y* = *x*), indicating that KBP‐E5 improved dose conformality and decreased modulation complexity compared to the CL [Figs. 6(a) and 6(b)]; however, the differences were not significant (*P *> 0.05). The median values of mIPCI for KBP‐E5 and the CL were 0.808 (range: 0.561–0.916) and 0.774 (range: 0.550–0.876), respectively. The median values of MCSv for KBP‐E5 and the CL were 0.078 (range: 0.040–0.204) and 0.071 (range: 0.042–0.176), respectively. Additionally, KBP‐E5 achieved a lower total number of MUs (median, 2,428.5 MUs [range: 1,836.4–4,054.7 MU]) than the CL (median, 2,532.3 MUs [range: 1,672.5–4,292.9 MU]) [Fig. 6(c)], although the difference was not significant (*P *> 0.05).

## 4 Discussion

To the best of our knowledge, this is the first study to assess the performance of KBPs in single‐isocenter VMAT for multiple brain metastases using the *k*‐fold CV method. The number of test samples was almost equivalent to previous reports on KB planning.^11^, ^12^, ^13^, ^14^, ^15^, ^19^ As shown in Table 3, most previous studies employed the hold‐out method; however, as described above, one disadvantage of this method is that the performance evaluation is subject to a higher variance given the smaller dataset, whereas multiple validations can be performed using the *k*‐fold CV method to conclusively determine the model overfitting. Using the *k*‐fold CV method, all of our KB models yielded clinically acceptable plans with a single optimization.

**Table 3:** Summary of previous reports on knowledge‐based planning. Most of the previous studies employed the hold‐out method.

	Disease	Training samples	Testing samples	Validation
Chang et al.^12^	Nasopharynx	79	20	Hold‐out
Hussein et al.^11^	Prostate	40	10	Hold‐out
	Uterus	37	10	Hold‐out
Chatterjee et al.^13^	Brain	82	21 (GBM)	Hold‐out
			24 (Others)	Hold‐out
Faught et al.^14^	Lung	30	20	Hold‐out
Yu et al.^15^	Liver	30 and 60*	13	Hold‐out
Babier et al.^19^	Oropharynx	216	1	LOOCV
This study	Multiple brain metastases	48	12	4‐fold CV

Abbreviations: CV, cross‐validation; LOOCV, leave‐one‐out cross‐validation, GBM = Glioblastoma.

* Note: two models were constructed.

KB planning, a machine‐learning tool for determining the best practice based on past successful treatment plans, creates KB models for improving the treatment plans for future patients. It is important to compare the performance of KB models in terms of generalization. In this study, *k*‐fold CV was applied for model evaluation and comparison. MA to MD were created with fixed parameters and applied to different patients’ groups for performance evaluation. The KB models were statistically equivalent after the adjustment of learned and fixed optimization parameters, although the interquartile ranges of the DVIs varied among them (Fig. 2). In the testing phase, the DVIs for ME were compared to the CL. We found that the ME generated statistically equivalent plans to the CL with a single optimization, except for PTV D_2%_. For hyperparameter tuning, which is equivalent to determining both mathematically learned objectives and fixed optimization objectives using RapidPlan^TM^, there is no clear rule that ensures the best performance. When comparing algorithms, statistical tests such as the McNemar test and Cochran’s Q test are commonly employed; however, it is impossible to obtain the “best” treatment planning algorithm because the definition of “best” varies according to clinical factors, such as the patient’s condition and treatment preferences. Therefore, we used the DVIs of the CL as the “best” outcome measures; the DVIs were compared between the KBPs and the CL for model evaluation.

According to a review paper on KB planning, several researchers achieved comparable, and often improved, VMAT plans using KBPs, while also reducing the planning time and variation in the plan quality.^9^ We demonstrated that the PTV D_2%_ for the KBP was significantly higher than that for the CL, while achieving the same dose conformality, and also that the radiation dose to the normal brain for the KBP was similarly low to that of the CL in patients with 2–18 PTVs, with a single optimization. In brain SRS, a higher D_max_ of the PTVs is associated with an improved local control of the dose, and a lower irradiated dose to the OARs with higher mIPCI values could decrease the radiation necrosis. According to previous research, a normal brain V_14 Gy_ is a good indicator of radiation necrosis in patients with large metastases after five‐fraction CyberKnife radiotherapy (Accuray, Sunnyvale, CA, USA)^20^; however, such dose‐volume constraints would not always be applicable, depending on the fractionation, target sizes, and the number of target volumes. During the manual inverse planning of single‐isocenter VMAT SRS for multiple brain metastases, it is difficult to set definitive clinical goals because of such variations. With KB planning, the realistically achievable dose distribution can be predicted and patients can receive high‐quality treatment even with limited time and human resources.

Numerous parameters reveal the plan complexity, such as MCSv, a modulation index, and the plan‐averaged modulation.^18^, ^21^, ^22^ The MCSv was employed in this study as it allows for an effortless comparison to other studies. In the study by Masi et al.,^19^ the MCSv values were in the range 0.25–0.50 for the conventional VMAT plans. Compared to this, our MCSv values for multiple brain metastases for the CL were extremely low (range: 0.042–0.176), indicating more complex MLC patterns compared to conventional VMAT plans. We found that the MCSv values were slightly higher for the KBP than in the CL [Fig. 6(b)] and fewer MUs were required [Fig. 6(c)]; thus, the KB models provided plans that are less intensity‐modulated than CLs.

Recently, the HyperArc (Varian Medical Systems) has been used clinically for multiple brain metastases. HyperArc plans consist of a maximum of three non‐coplanar arcs, based on four of five possible fixed angular couch positions to be selected between (0°, ±45°, +90°) and (0°, ±45°, −90°), with each arc having a fixed length of 180° and an automatically selected collimator angle. Several studies have demonstrated the superiority of the HyperArc to single‐isocenter VMAT in terms of the conformity and dose falloff.^23^, ^24^ In this study, the same beam parameters (beam gantry, couch, and collimator angles) used in an approved CL were employed during the testing phase; however, in a clinical situation, the beam arrangement is determined through a process of trial and error, which is time consuming and affects the plan quality. As shown in the present and previous studies,^23^, ^24^ both KB planning and HyperArc are effective in reducing doses to OARs while maintaining the target coverage for multiple brain metastases. Both methods have advantages and disadvantages with respect to the optimization of parameters including the number of arcs, collimator angle, couch angle, and arc lengths; therefore, an approach combining the advantages of KB planning and HyperArc would provide more conformal dose distributions in a shorter planning time.

There were some notable limitations to this study. First, this study was conducted retrospectively, and the KB models employed were derived from the data of a single institution. To assess the applicability of our model, the clinical data of other institutions, or of different cohorts and beam arrangements, should be used prospectively for external validation. Second, eligible patients can be treated safely without any special effort to decrease the irradiated dose to OARs other than the brain. Patients with a large target volume, prior history of cranial irradiation, or a lesion located in or close to brainstem are treated with other fractionations in our institute; such cases were not included in this study.

## 5 Conclusions

We employed fourfold CV for the evaluation and selection of KB models. Statistically equivalent models (between the CL and final KB model) were generated after the adjustment of learned and fixed optimization parameters. After confirming the generalization performance of the models, the final KB model was applied to the test group. We demonstrated that the KB model in the single‐isocenter VMAT for multiple brain metastases was equivalent in dose distribution, MCSv, and the total number of MUs to the CL with a single optimization.

## Consent for publication

The consent for publication was obtained via our institution’s form.

## Authors’ contributions

NK performed the planning study and statistical analysis, and drafted the manuscript. NK, MN, HH, NM, KT, and MU conceived the study, participated in its design and coordination, and helped to draft the manuscript. All authors read and approved the final manuscript.
